# Bio-Inspired Functional Surface Fabricated by Electrically Assisted Micro-Embossing of AZ31 Magnesium Alloy

**DOI:** 10.3390/ma13020412

**Published:** 2020-01-16

**Authors:** Xinwei Wang, Jie Xu, Chunju Wang, Antonio J. Sánchez Egea, Jianwei Li, Chen Liu, Zhenlong Wang, Tiejun Zhang, Bin Guo, Jian Cao

**Affiliations:** 1 Key Laboratory of Micro-Systems and Micro-Structures Manufacturing, Ministry of Education, Harbin Institute of Technology, Harbin 150080, China; xinweiwang@hit.edu.cn (X.W.); xjhit@hit.edu.cn (J.X.); 13B909069@hit.edu.cn (J.L.); bguo@hit.edu.cn (B.G.); 2 Laboratory for Space Environment and Physical Sciences, Harbin Institute of Technology, Harbin 150001, China; 3 School of Mechanical Engineering, Harbin Institute of Technology, Harbin 150001, China; wangzl@hit.edu.cn; 4 School of Mechanical and Electrical Engineering, Robotics and Microsystems Center, Soochow University, Suzhou 215131, China; 5 Department of Mechanical Engineering, Universitat Politècnica de Catalunya, Av. Eduard Maristany, 16, 08019 Barcelona, Spain; sanchezegea.antonio@gmail.com; 6 Beijing Hangxing Machinery Manufacture Limited Corporation, Beijing 100013, China; 7w7z@sohu.com; 7 Department of Mechanical Engineering, Northwestern University, 2145 Sheridan Rd, Evanston, IL 60208, USA; jcao@northwestern.edu

**Keywords:** electrically assisted, micro-embossing, bio-inspired functional surface, bulk metallic glass, photolithography

## Abstract

Developing bio-inspired functional surfaces on engineering metals is of extreme importance, involving different industrial sectors, like automotive or aeronautics. In particular, micro-embossing is one of the efficient and large-scale processes for manufacturing bio-inspired textures on metallic surfaces. However, this process faces some problems, such as filling defects and die breakage due to size effect, which restrict this technology for some components. Electrically assisted micro-forming has demonstrated the ability of reducing size effects, improving formability and decreasing flow stress, making it a promising hybrid process to control the filling quality of micro-scale features. This research focuses on the use of different current densities to perform embossed micro-channels of 7 μm and sharklet patterns of 10 μm in textured bulk metallic glass dies. These dies are prepared by thermoplastic forming based on the compression of photolithographic silicon molds. The results show that large areas of bio-inspired textures could be fabricated on magnesium alloy when current densities higher than 6 A/mm^2^ (threshold) are used. The optimal surface quality scenario is obtained for a current density of 13 A/mm^2^. Additionally, filling depth and depth–width ratio nonlinearly increases when higher current densities are used, where the temperature is a key parameter to control, keeping it below the temperature of the glass transition to avoid melting or an early breakage of the die.

## 1. Introduction

Nature has developed biological surfaces with periodic multiscale arrangements (macro/micro/nano levels), showing a high state of intelligent functionality. One example of these is the shark skin, which has placoid scales, with a rectangular base implanted in the skin with small spines or bristles that emerge to the surface. This particular scale configuration keeps the skin clean, and also reduces drag [1,2]. In recent years, researchers have fabricated many bio-inspired functional surfaces by using different materials and processes to achieve multiple intelligent performances, such as antibiofouling, super-hydrophobicity and wear-resistance. In particular, Chen et al. [2] made an overview of the bio-inspired sharkskin surface in terms of drag reduction mechanism, fabrication methods and applications. Sharklet AF^TM^ [3] developed silicon wafers by using photolithography that was also coated with a reactive ion etching. This reactive coating transferred defence properties to the elastomers against bacteria [4]. Accordingly, micro-manufacturing processes, like photolithography, micro-machining, laser ablation, direct 3D printing, micro-molding and micro-embossing, are commonly used for bionic microstructure fabrication, although each of these processes presents some difficulties. Photolithography is a complex and a high cost process, which transfers relatively poor mechanical capabilities to silicon. In micro-machining, micro-machining tools are easy to wear during cutting micro-patterns. Laser ablation achieves micro-features with high accuracy, despite being a low efficiency and high cost process in terms of energy consumption. Similar to laser ablation, direct 3D printing could produce very complicated patterns with multiscale features, but is time-consuming and limited in materials [1,2]. Finally, micro-molding is an economic process to obtain bionic microstructures by using organic polymers, although poor mechanical properties and easy aging are denoted in the dies.

Micro-embossing based on plastic deformation is a simple and highly efficient method to mass produce bio-inspired functional surfaces on different types of materials, including metals. This process presents several advantages: it has a high strength, low cost and is environmentally friendly. The most relevant issue to address when using micro-embossing is size effects, which can cause filling defects and breakage of micro-features on the embossed dies. Cao et al. [5] embossed micro-channel patterns with 20 µm depth and 100 µm width on 500 µm thick AA5052 sheets by micro-rolling-based surface texturing. They concluded that the relative velocity between the upper and lower rollers significantly affected the filling ability. Also, Wang et al. [6] investigated the effects of grain size and cavity width on the filling ability of pure nickel during micro-embossing. They observed that the worst filling ability occurred for a width of 50 μm and ratio of cavity of 1.04, which was attributed to the coupling effect of grain size and cavity dimension. Sareh [7] studied a double corrugation surface (origami pattern) with the aim to analyze the flat-foldability depending on the surface characteristics. They found the interrelation of crystallography and computational geometry for designing complex origami structures. Also, Le and Goo [8] analyzed the thermomechanical impact of thermal layer protection based on bio-inspired, corrugated-core sandwich structures. These sandwich structures improved the deflection capabilities and reduced weight up to 65% compared with non-textured structures. Qiao et al. [9] remonstrated the poor filling quality and high damage rate of micro-features of the dies when performing embossed micro-channel of 10 μm in Al-1050 at room temperature. On the contrary, a relatively good quality of micro-channel patterns was obtained when embossing ultra-fine-grained material at elevated temperatures. Consequently, a possible way of reducing the size effect on filling quality is to increase forming temperature and improve plastic flow based on superplasticity, but a relatively longer processing time would be required with traditional thermal-assisted processes. In order to reduce this processing time, researchers [10] found that the passage of electricity during plastic deformation would give rise to multiple alterations of forming property and microstructure. For example, Tang et al. [11] made a comprehensive review of the electrically assisted (EA) forming processes including EA drawing, EA rolling and EA punching, which consistently proved that an electric current could reduce deformation resistance, improve plasticity, simplify processes, increase energy efficiency, lower cost and increase time-effectiveness. However, it should be noted that few works have focused on the EA micro-forming. Recently, Wang et al. [12] found that current-induced softening increases with decreasing grain size but increasing specimen size. Other researchers [13,14] concluded that localized Joule heating at the grain boundaries would affect the mechanical properties, making the Hall–Petch effect smaller as compared to the electrical/thermal decoupling tests. This assumption was demonstrated by Cao et al. [15], where local intergranular cavitation and local grain boundary melting were observed in tensile samples subjected to an electric current. Additionally, Lai et al. [16] fabricated micro-channels on 316 L stainless steel sheets by using EA micro-embossing. The results showed that the channels were deeper and the residual stress was smaller when the process was electrically assisted. Finally, Cao et al. [17] had also induced an electric current during a micro-rolling surface texturing process, in order to increase the depth–width ratio of micro-channels. They found that the channel depths of AA3003-H14 and Ti6Al4V increased by 15% and 200%, respectively. These changes were attributed to the difference in electrical resistivity and, consequently, the Joule heating effect, which are much higher in titanium alloy.

Considering the possible potential of controlling the filling quality and the difficulty of forming bio-inspired functional surfaces with high aspect ratios on metals, especially for difficult-to-form materials with high anisotropy [18], this research focuses on investigating the EA micro-embossing process in AZ31 magnesium alloy. This hybrid process tries to fabricate micro-channels and sharklet patterns in magnesium alloy when using dies manufactured with photolithographic silicon-molding, thermoplastic-forming of bulk metallic glass (BMG) and EA micro-embossing. Then, the EA’s capabilities regarding the filling ability of bio-inspired micro-features are investigated. As a result, these EA textured surfaces were well transferred from BMG dies to large AZ31 areas, achieving micro-features down to ~2 µm and a depth–width ratio up to ~1.4.

## 2. Methodology

### 2.1. Sample Preparation

**Figure 1:**
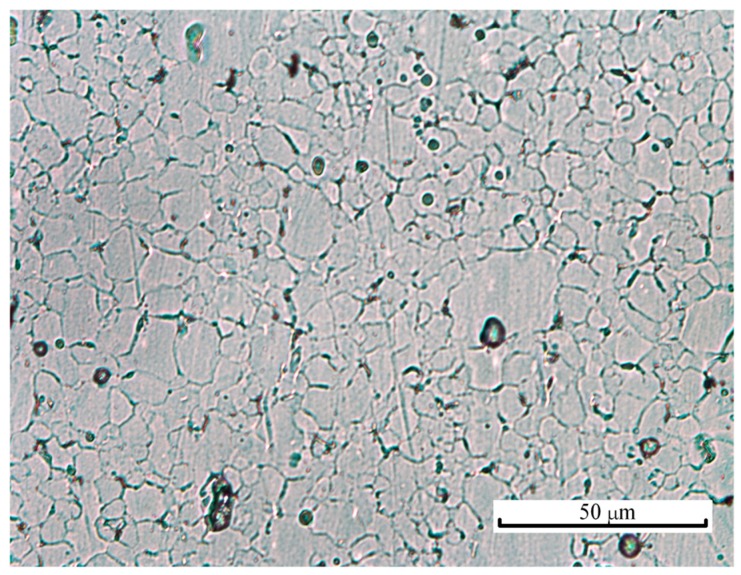
Optical micrograph of the studied AZ31 magnesium alloy to determine the grain size.

**Figure 2:**
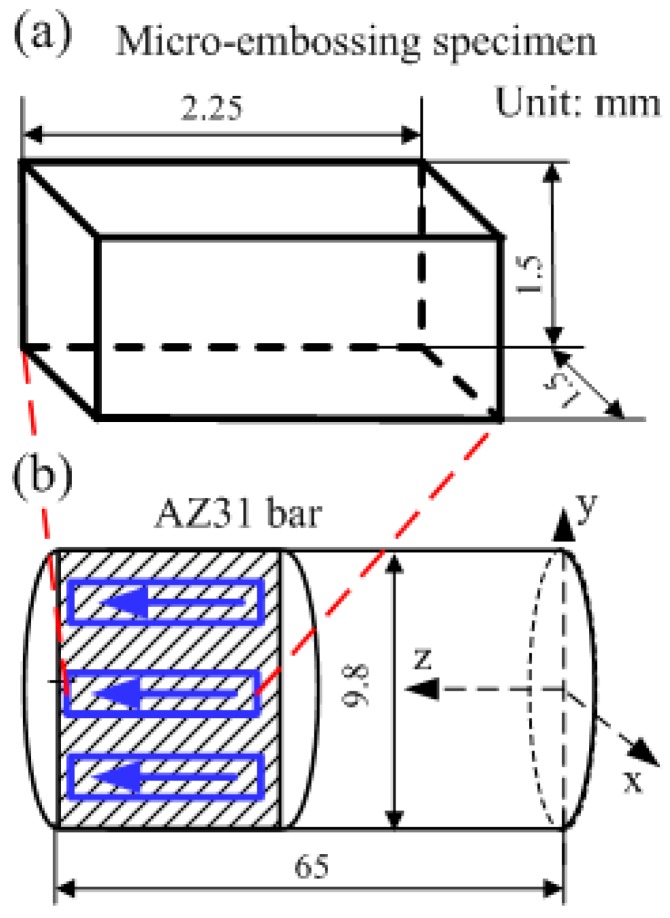
Schematic illustration of specimen preparation by using a CNC milling machine: (**a**) the geometry of the micro-embossing sample; (**b**) the sample cutting section and direction.

The material used in this research was a commercial drawn AZ31 magnesium alloy rod with a diameter of 15 mm and the following chemical composition: 94.8 wt% Mg, 3.5 wt% Al, 1.2 wt% Zn and 0.5 wt% Mn. The as-received materials were annealed at 400 °C for 2 h, which brought equiaxed grains with an average size of 7.1 ± 1.1 μm. Figure 1 shows the material microstructure after the procedures of grinding, polishing and etching. Micro-embossing specimens were cut into cuboid shapes with the height (*H*) and the base side length (*L*) of 2.2 and 1.5 mm, respectively. A precision CNC milling machine was used to cut the specimens from the axial cross sections of the annealed rods along the axial direction, as shown in Figure 2. Note that the machining tolerance for all the geometric dimensions was ±0.1 mm. Both end surfaces of the samples were mechanically polished using sandpaper to avoid surface oxidation prior to testing, since bio-inspired micro-features were embossed on the end surfaces.

### 2.2. Preparation of Micro-Channel and Sharklet Dies

Micro-channel and sharklet pattern dies were fabricated by integrating the use of the properties of BMGs, photolithography, BMGs’ crystallization and thermoplastic forming (TPF) in supercooled liquid region (SCLR). Specifically, silicon dies with micro-channel and sharklet patterns were first fabricated using photolithography. Afterward, we compressed BMGs on the silicon dies with TPF to make our BMG dies. These two operations are thoroughly described below.

**Figure 3:**
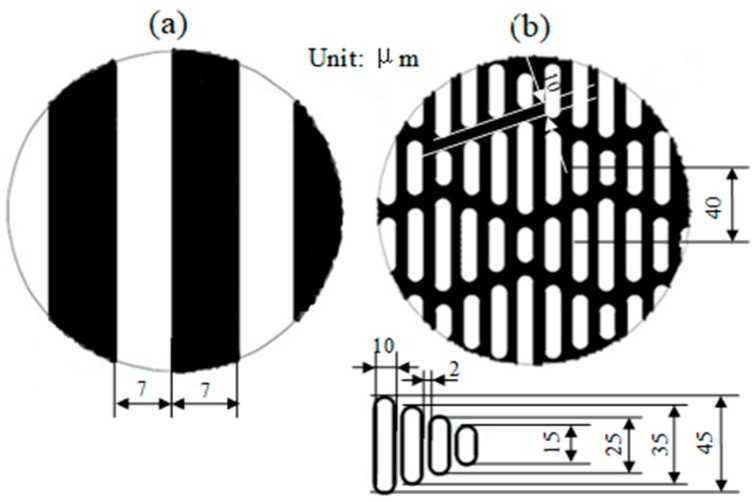
Photomask designs: (**a**) the micro-channel and (**b**) the sharklet patterns.

*(1) Photolithographic Silicon Molding:* In this study, the silicon dies with micro-channels and sharklet patterns were prepared at Northwestern University, Evanston, IL, USA. A deep reactive ion etching with an etching rate of ~3.6 µm/min was deployed for 6 min. Figure 3a,b exhibit the photomask designed features of the micro-channel and sharklet patterns, respectively. The white areas in Figure 3 had no photoresist covering, and were etched away to produce depth channels of 15–20 µm. Through a series of photolithographic procedures, such as cleaning, coating, exposure, developing, etching and photoresist removal, multiple silicon molds were made to have large areas of micro-channel patterns, as well as sharklet patterns. Table 1 lists the average depth and width dimensions, measured at five points on the silicon molds by using Alicona Infinite Focus-Optical 3D measurement and inspection. The average depth and width for the sharklet pattern were very close to our designed features. Note that the design of the micro-channel width was 7 µm, although the silicon molds had widths of 8–9 µm. This dimensional dispersion can be associated with the leaking and scattering of the UV light caused by dust between the wafer and mask during the exposure process.

*(2) TPF of BMG dies:* BMGs are known to have dramatic softening when reheated into the SCLR (between the glass transition temperature *T_g_* and the crystallization temperature *T_x_*), where the glass relaxes into a metastable liquid before its eventual crystallization. This behavior allows to BMGs to have a high viscosity, high formability and be insensitive to heterogeneous influences, which is comparable to plastics [19]. As a result, many TPF-based processes [20] were developed for BMGs in SCLR to achieve high dimensional accuracy in complex geometries, especially in the micro-forming industry [21,22].

**Figure 4:**
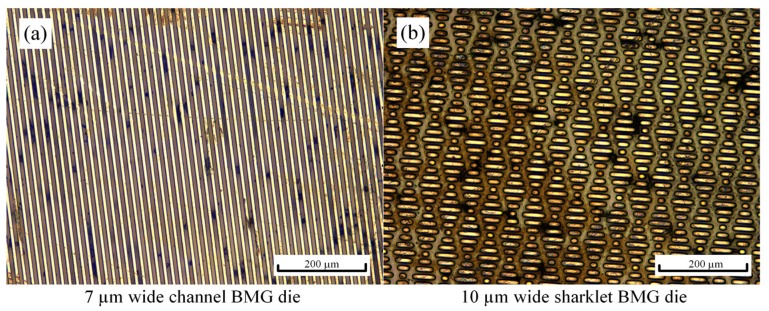
Characterizations of the textured bulk metallic glass (BMG) dies: (**a**) the micro-channels and (**b**) the sharklet patterns.

Zr_35_Ti_30_Cu_8.25_Be_26.75_ was selected as the as-received material, since it has a small *T_g_* and a large SCLR, i.e., Δ*T* = *T_x_* − *T_g_* = 159 K, for any known commercial BMG. The thermal, mechanical and rheological properties of Zr_35_Ti_30_Cu_8.25_Be_26.75_ are listed in Table 2. TPF generally occurs at temperatures above *T_g_* and below *T_x_* for glassy material, to apparently decrease flow stress and avoid crystallization. The fabrication of the bio-inspired functional surface on BMGs based on TPF includes pre-compression, polishing and thermoplastic embossing. Pre-compression of small pieces of round BMG specimens was conducted at 673 K for several seconds under 1000 N by using an Instron compression machine. Afterward, each pre-compressed round specimen was polished with 1 μm polishing slurry to obtain a smooth and parallel surface prior to embossing bio-inspired micro-features. The thickness of the pre-compressed BMG samples was around 0.6 mm. Thermoplastic embossing of BMGs when using the 7 μm micro-channel, and 10 μm sharklet pattern of silicon molds was performed at 700 K for 30 s under 3200 and 4300 N, respectively. After that, the BMG dies with bio-inspired functional surfaces were obtained by etching out the silicon set in micro-features in 20% KOH solution at 120 °C. Micro-channels and sharklet features on the fabricated BMG dies were characterized, as shown in Figure 4. The figure shows well-ordered micro-scale patterns over large areas fabricated on Zr_35_Ti_30_Cu_8.25_Be_26.75_ BMG samples using the TPF-based processes. The micro-scale features were measured at least three times, e.g., the micro-channel BMG die has 7.07 ± 0.27 µm of width and 15.28 ± 0.3 µm of depth with a depth–width ratio over two; the micro-sharklet BMG die has 15.62 ± 1.21 µm of width and 8.88 ± 0.33 µm of depth, with a depth–width ratio below one. These results show that it is harder to emboss the sharklet micro-feature, perhaps because the deformation state tends to be more complicated due to the complex sharklet pattern, impeding plastic flow.

### 2.3. EA Micro-Embossing Test

**Figure 5:**
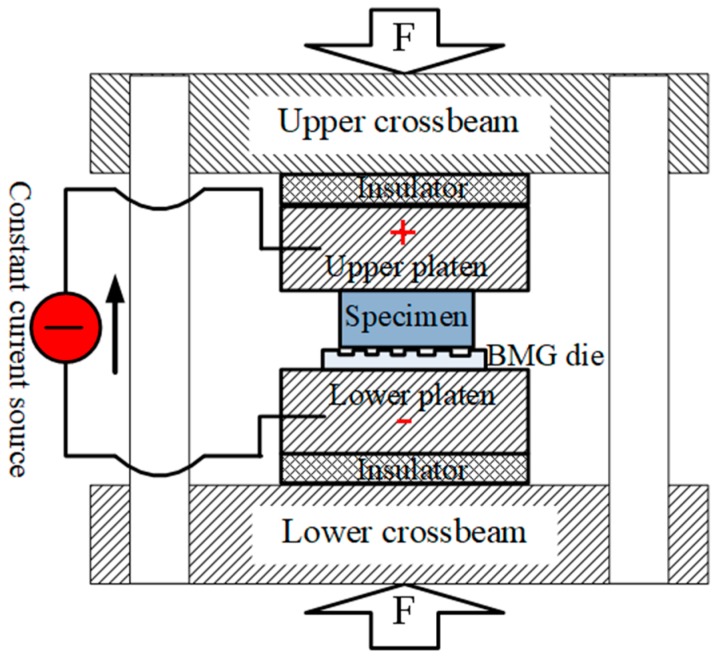
Schematic illustration of the electrically assisted (EA) micro-embossing system.

The EA micro-embossing system was schematically shown in Figure 5. The BMG dies were placed on the lower platen of the micro-embossing machine with the bio-inspired functional surface upward. Then, the cuboid specimen was placed on the BMG dies with the polished surface contacting the bio-inspired micro-scale patterns. During micro-embossing, the upper and lower crossbeams were driven by a DC motor to move downward and upward, respectively. The embossing force was measured by a load cell of 1000 N capacity with a resolution of 0.1 N. Similar to in [23], a rectifier-based DC power supply with a maximum output current intensity of 300 A was used to pass a continuous constant current through the specimen, which was insulated from the loading system. The temperature was measured by an infrared camera located on the back side of the sample. A layer of black paint was added on the back surface prior to tests to set the emissivity and reduce the temperature errors with the infrared camera. Furthermore, different current densities were chosen (i.e., 0 A/mm^2^, 6 A/mm^2^, 10 A/mm^2^, 13 A/mm^2^) to study the current induced effect on the fillability of bio-inspired micro-features. All the tests were conducted with a fixed strain rate of 0.01 s^−1^ for a duration of ~50 s and stopped after reaching ~300 N. All the workpieces were cleaned with acetone in an ultrasonic cleaning tank after the EA micro-embossing tests.

## 3. Results and Discussion

**Figure 6:**
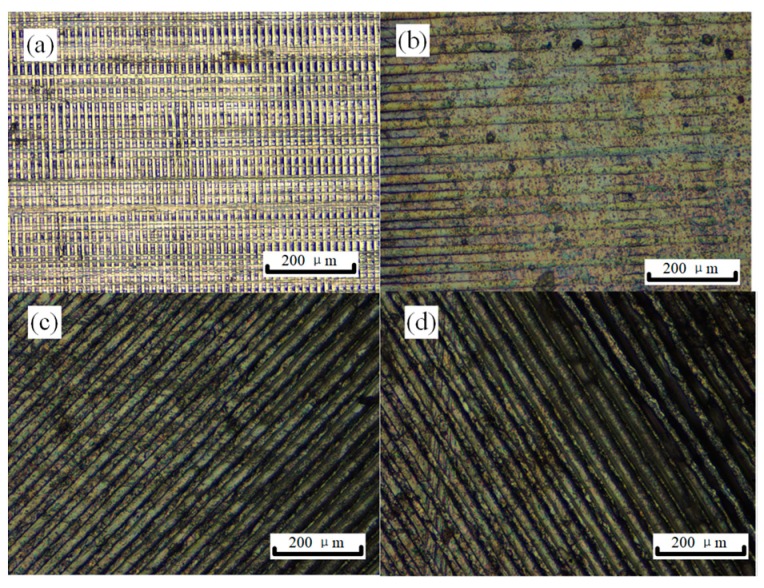
Micrographs of the micro-channel patterns obtained by EA micro-embossing at different current densities: (**a**) 0 A/mm^2^, (**b**) 6 A/mm^2^, (**c**) 10 A/mm^2^, (**d**) 13 A/mm^2^.

**Figure 7:**
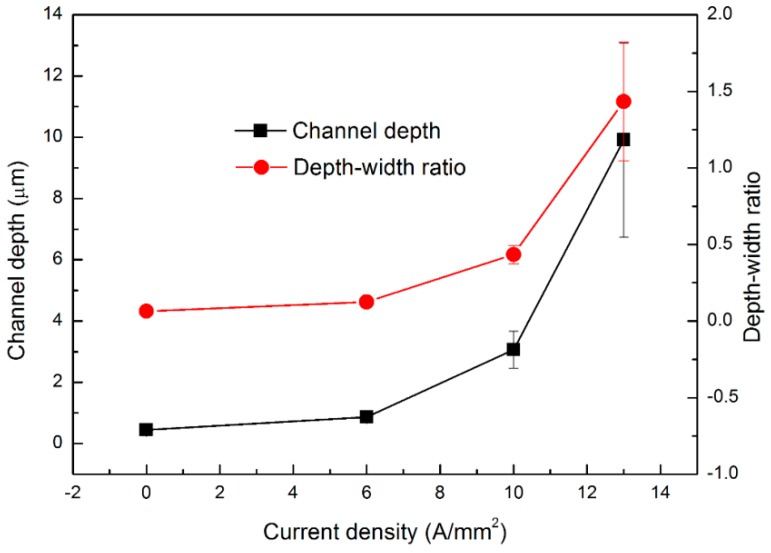
Variations in channel depth and depth–width ratio with respect to different current densities.

Figure 6 shows four embossed areas performed with different current densities in AZ31 magnesium alloy. As can be seen, the channel pattern transferred from the BMG dies is disorderly at 0 A/mm^2^, with the micro-feature size probably approximate to the metal cutting error. During EA micro-embossing, it is observed that the geometry integrity of the micro-channel pattern on the sample surfaces is becoming more accurate with the increase in current density. Note that the channel width is not uniform, probably because Joule heating tends to concentrate at a few local areas of the embossing interfaces. Accordingly, the non-uniform distributions of current density, plastic deformation and uneven contact/friction favors local melting areas between channels. The channel depth and width were measured at least three times in each sample for repeatability purposes. The results also exhibit that the channel width is ~7 μm, which is very consistent with that of the BMG dies. Figure 7 shows the relationship of the channel depth, depth–width ratio and current density. This figure denotes the non-linear increase in the channel depth with respect to current density, showing a faster increasing rate at the higher current density. The variation in depth–width ratio with respect to the current density also exhibits a lower convex relationship. An unexpected result for the embossed depth was found, i.e., a depth 20 times higher at 13 A/mm^2^ compared to 0 A/mm^2^. An area of channel patterns with a depth-width ratio of ~1.4 was obtained (exceeding the common ratio of ~1 in a traditional micro-embossing at a width far below 50 μm [6]). Note that the channel depth does not increase much for current densities lower than 6 A/mm^2^, indicating that there also exists a current density threshold [12] during EA micro-embossing. The Joule heating temperature also non-linearly increases with the current density, which was found to be <200 °C (below the glass transition temperature of the BMG without damage of the BMG micro-patterns). Consequently, it resulted in a faster softening and a better filling ability for the embossing samples.

**Figure 8:**
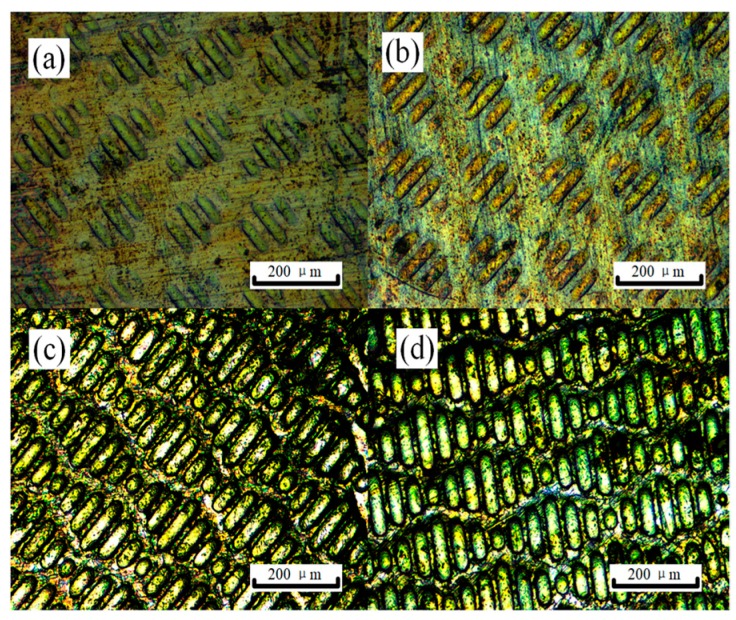
Micrographs of the sharklet patterns obtained by EA micro-embossing at different current densities: (**a**) 0 A/mm^2^, (**b**) 6 A/mm^2^, (**c**) 10 A/mm^2^, (**d**) 13 A/mm^2^.

**Figure 9:**
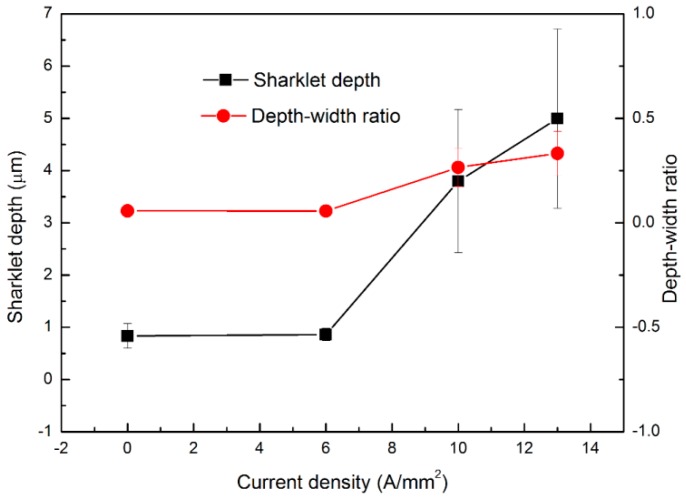
Variations in sharklet depth and depth–width ratio for different current densities.

Figure 8 exhibits the sharklet patterns on AZ31 sample surfaces transferred from BMG dies for different current densities. Only a few parallel ridges with different lengths were marked into the workpieces during micro-embossing without applying an electric current. Similar to the observations in Figure 6, the passage of an electric current during micro-embossing improves the geometry integrity of the sharklet patterns and fillability, particularly for higher current densities. Also, the sharklet feature copied from BMG dies has a high repeatability over the sample surface when using current densities above 10 A/mm^2^ (the threshold was found at 6 A/mm^2^). A similar result is observed in Figure 9, where the embossed sharklet depth nonlinearly increases with current density and the width varies in a smaller order. The depth–width ratio in Figure 9 is found to not change with the current density as much, as compared to the micro-channel embossing, e.g., ~0.33 at 13 A/mm^2^ vs. 0.06 at 0 A/mm^2^ during shark embossing. These differences could be attributed to the enhanced material flow resistance caused by the complex micro-ridges/channels in different directions. The increasing rate of sharklet depth against current density also rises over 6 A/mm^2^, considered as the current density threshold where the electric current starts to significantly improve the embossing fillability. The Joule heating temperature is relatively consistent with that in the EA micro-embossing of channel patterns, i.e., below 200 °C (below the glass transition temperature of the BMG without damage of the BMG micro-patterns). An excessive Joule heating would occur above a current density, e.g., ~20 A/mm^2^, giving rise to a sharp deterioration in the embossed texture quality due to die breakage/melting.

## 4. Conclusions

In this research, typical bio-inspired functional surfaces with a micro-channel of 7 μm and sharklet patterns of 10 μm were EA micro-embossed on AZ31 magnesium alloy. To do that, textured BMG dies prepared by TPF-based compression on photolithographic silicon molds were used. We found that both the filling depth and depth–width ratio non-linearly increased with current density. The results also showed that larger and accurate areas of channel and sharklet textures could be fabricated by using EA micro-embossing. The smallest feature, down to ~2 μm, and a depth–width ratio of up to ~1.4 were addressed when using current densities of 13 A/mm^2^ and temperatures below the glass transition of the BMG. Finally, the geometry integrity of bio-inspired functional features could not be well obtained below 6 A/mm^2^, indicating that the threshold of current density occurs in this EA micro-embossing process.

## Figures and Tables

**Table 1:** Geometrical measurements of depth and width of the silicon molds.

Silicon Die	Avg. Depth (µm)	Avg. Width (µm)	Stdev. of Width (µm)
Channel pattern	20.13	8.03	0.95
Sharklet pattern	19.94	10.09	1.43

**Table 2:** Thermal, mechanical, and rheological properties of Zr_35_Ti_30_Cu_8.25_Be_26.75._

Glass Transition Temperature *T_g_* (K)	Crystallization Temperature *T_x_* (K)	Liquidus Temperature *T_l_* (K)	Angell Fragility *m*	Thermal Stability *S*	Shear Modulus *G* (GPa)	Poisson Ratio *v*
578	737	1044	65.6	0.34	31.8	0.37
